# Nurse Education and Mathematical Competency: Implementation of an Online, Self-Directed, Prerequisite Model

**DOI:** 10.3390/ijerph182413106

**Published:** 2021-12-12

**Authors:** Daniel H. Jarvis, Karey D. McCullough, Tammie R. McParland

**Affiliations:** 1 Schulich School of Education, Nipissing University, North Bay, ON P1B 8L7, Canada; 2 School of Nursing, Nipissing University, North Bay, ON P1B 8L7, Canada; kareym@nipissingu.ca (K.D.M.); tammiem@nipissingu.ca (T.R.M.)

**Keywords:** nursing education, mathematics, dosage calculation, curriculum, technology, online learning

## Abstract

Mathematical competency in the profession of nursing has increasingly become a central focus as more nursing students appear to struggle with basic concepts of arithmetic, mental estimation, and critical reasoning. This paper highlights how one School of Nursing in Ontario, Canada implemented a Dosage Calculation Competency Test model which involved an online, self-directed, prerequisite approach to improve student mathematical competency and confidence. The purpose of this research case study was to document, through shared participant perceptions, the creation, implementation, and subsequent modifications to a Dosage Calculation Competency Test model in light of student needs and advances in online learning and assessment. The research design combined a quantitative survey of Year 1–4 nursing students, followed by a series of qualitative, semi-structured interviews with nursing students and program instructors. The study took place within a School of Nursing undergraduate program in Ontario, Canada. Forty-four participants, including students from all four years of the nursing program, completed the survey, followed by individual interviews with nine students and six faculty instructors. Survey (the open-response items) and interview data were analyzed thematically using *ATLAS.ti* (ATLAS.ti, Berlin, Germany). The authors recount the new DCCT model’s development, implementation, and subsequent modifications and further discuss student/instructor perceptions of learning types, math confidence, and competency. The paper concludes with a series of seven key recommendations for nursing programs.

## 1. Introduction

Over the past few decades, a disturbing phenomenon has emerged within undergraduate nursing education. Nursing students are increasingly demonstrating an overall lack of basic, as well as more advanced, math skills [1]. The ability to demonstrate mathematical competency as a nurse remains a key component of professional practice, even in the face of the technological paradigm shift happening in healthcare. Unsafe medication usage is the leading cause of medical harm in the world [2]. Nurses are responsible to prepare, administer, and evaluate medications in a wide variety of healthcare contexts. Medication administration is a cornerstone of most schools of nursing and is included in standards of practice for nurses at the regulatory [3], as well as the institutional level (e.g., hospitals, community care homes).

Nurses require a set of mathematical skills in order to safely administer various types of medications (e.g., oral tablets and fluids, injections, intravenous) to the patients in their care. Part of this mathematical calculation competency involves basic mathematical knowledge such as working with fractions, ratios, and rates, as well as being proficient with conversions between unit measures (e.g., milligrams to grams, teaspoons to tablespoons) and systems (Imperial to metric). Nurses are central to medication safety since they are predominantly responsible for the administration of patient medication in a variety of healthcare contexts including acute care settings [4]. 

The intersection of nursing and mathematical competency has become an increasing concern among nurse educators and health administrators. Nursing students with relatively weak math skills are enrolling in undergraduate programs, and, after several years of study, appear to often still be lacking the mathematical competence and self-confidence required for practice [5]. Math anxiety, math phobia, or even clinical dyscalculia (sometimes called “number dyslexia) can often contribute to the high levels of stress associated with calculation skills and related assessment [6]. Diagnostic tools were developed to provide nursing programs with an effective way to accurately identify and address curricular weaknesses related to dosage [7]. 

In order to address this lack of mathematical competency among students, undergraduate nursing programs have implemented a number of different strategies to address this important issue. Requiring the completion of certain secondary school math/science courses [8], achieving certain grades in these courses [9], and administrating a math proficiency entrance exam at the university level [10] serve as examples of such strategies. Many nursing education programs have implemented some form of a mandatory, high-stakes drug calculation test [11], i.e., a course, or series of courses, that a nurse must take prior to graduating and ultimately becoming formally “registered” by a regulatory body. Interestingly enough, nursing students, even when successful in adequately passing their compulsory math/dosage tests, often indicate that they still feel a lack of confidence in their perceived drug administration skills within clinical settings.

Another way to improve mathematical competency among undergraduate nursing students is to provide more consistent and competent clinical instruction [4,12]. Some contend that the dosage testing must be carried out within a clinical practice context in order to be authentic and truly beneficial for students [13,14]. However, other researchers have argued the opposite case, highlighting the potential effectiveness of online instructional formats in realizing the goal of increased student achievement regarding mathematical competency [1,15]. A number of researchers make the case for a multi-faceted or more *balanced* approach, calling for an amalgam of strategies that include both technology-enhanced and face-to-face components [16,17]. 

Recognizing increasing student weakness in mathematical competency, and that dosage calculation skills were being taught in a variety of ways and with different results within their program, a School of Nursing in Ontario, Canada reviewed its curriculum and determined that moving to a comprehensive and more standardized method to teach math competency was needed to increase student achievement and confidence in this vital area. A new Dosage Calculation Competency Test [DCCT] model was introduced over the period of several years, and this involved an online, self-directed, and prerequisite approach. In the remainder of this paper, we outline the rationale for, and issues relating to, the transition from the previous model to the existing DCCT model; present findings based on a case study involving an online survey and subsequent student/instructor interviews; and offer a series of related recommendations for schools of nursing that may be contemplating similar changes to their program.

## 2. Methods

The researchers framed the investigation around the following two research questions (RQs): 

RQ1: What was the rationale for the transition from a teacher-directed, on-site, course-embedded DCCT model to a self-directed, online, prerequisite model; how was it implemented; and what modifications were later made to the model?

RQ2: According to nurse student and instructor perceptions, to what degree, and in what ways, is the DCCT model adequately preparing students for nursing practice?

The case study was designed and implemented according to qualitative case study standards [18,19,20], and featured the following components: (i) a review of the literature focusing on nursing education/curriculum and mathematical skills (most specifically relating to dosage calculations); (ii) the design and implementation of an online survey for nursing students (Years 1–4 within a Collaborative Bachelor of Science in Nursing (BScN) program); and (iii) semi-structured interviews with a sub-set of participant volunteers, based on their comments made within open-response survey questions, along with a group of solicited nursing program instructors and administrators who were previously and/or currently involved with the DCCT program. The research study protocol was approved by the university’s Research Ethics Board (REB) and all participants signed letters of informed consent. The online survey (Appendix A) was created using licensed and secure *Qualtrics* software (Qualtrics, Provo, UT, USA). The survey collected answers to 22 direct questions (both descriptive and Likert scale responses), as well as approximately 5000 words of feedback by way of the final three open-response item questions. 

**Figure 1:**
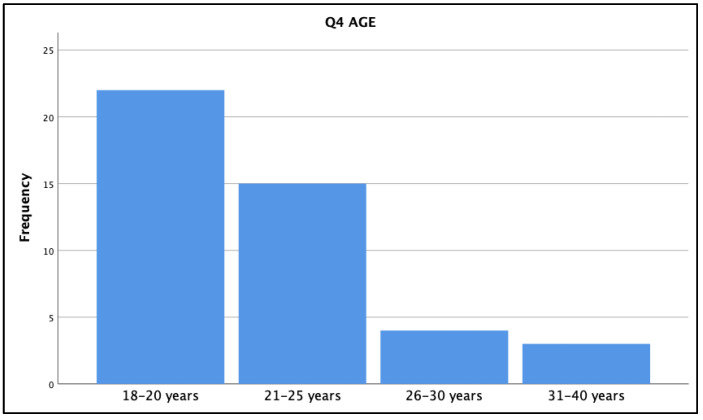
BScN Collaborative Program survey participants (*n* = 44) by age bracket.

All of the Year 1–4 nursing students in the Collaborative BScN program (i.e., a joint university/college program) were invited to participate in the online survey (see Appendix A). Of the approximately 400 current students within the program, 44 agreed to complete the survey (i.e., an 11% response rate) by following an embedded link within the emailed invitation. Five of the participants were part of the 3-Year version of the Collaborative program (i.e., admitted after completing a college-level nursing diploma); 39 were part of the 4-year version of the Collaborative program. All four years of program study were represented in the participating group, with students from Year 1 (11), Year 2 (8), Year 3 (14), and Year 4 (11) all offering their insights via the online survey. Our participants also reflected a cross-section of ages (18 were “18–20 years of age,” 15 in “21–25 years, 4 in “26–30 years,” and 3 in “31–30 years” (see Figure 1), and in terms of declared gender, 37 of our research survey participants were female and 7 were male.

From the 44 survey participants, 9 nursing students agreed to take part in subsequent interviews. Instructors and administrators who had previously been, or currently were, associated with the Dosage Calculation Competency Test (DCCT) model were also invited via email to take part in an interview. Thus, fifteen interviews (two Year-1 students; one Year-2 student; three Year-3 students; three Year-4 students; and six faculty members, five of which had served in both instructional and administrative roles over the time period in focus) were conducted and audio-recorded from December 2019 through April 2020, with each interview lasting approximately 40–60 minutes (see Appendix B). The interviews were recorded using *Zoom* video-conferencing software (Zoom, San Jose, CA, USA), then were transcribed by a graduate student and returned to participants for member checking of the content for accuracy. The interview transcription data, over 70,000 words (140 pages), were then analyzed using *ATLAS.ti* software.

All of the collected open-response survey data and interview data were entered into *ATLAS.ti* qualitative software and careful thematic analysis using multi-layer coding was conducted. From a large list of original codes, several overarching themes emerged as being key to the narrative of the new DCCT Model adoption history, as well as a second set of themes relating to key issues surrounding nurse perceptions and practice. Limitations of the study included the response rate (11%) which provided adequate data for our purposes but could have been higher; the fact that only single interviews were possible with our participants due to time constraints; and the lack of access to comparable student achievement data from before and after the DCCT model implementation. 

## 3. Results

In this section, we will address Research Question 1 in light of the collected participant data relating to the following themes: the rationale for making changes to the previous Dosage Calculation Competency Test (DCCT) model; the implementation of the new model; and the various subsequent modifications that were made to the new model.

### 3.1. Rationale for Change

Prior to the transition to the new DCCT model that took place during the years 2012–2014, nursing students in the Collaborative Bachelor of Science in Nursing program would learn about dosage calculations as part of general nursing courses that each involved on-site classroom attendance, simulation labs, and clinical (hospital/community) placements. In the previous model, each clinical course was a pass/fail course requiring an overall Satisfactory completion designation in order for a nursing student to progress through the program. Nursing students who were not successful in obtaining the 90% baseline achievement in their first writing of the DCCT were usually provided with remedial support and then allowed to attempt the DCCT assessment again. As one instructor noted: “If they failed that second test attempt, typically they were at risk for failing out for the semester.... They did let them go into clinical, but they could not administer meds” (P01). Noting the increasing number of students rewriting the math tests, with some also failing the math component of the clinical course, and hence the course and program, the nursing faculty understood that they had a significant and recurring problem on their hands.

While the previous model did serve to highlight the importance of mathematical competency for nursing practice by including the math skills content within the clinical courses, and by requiring successful completion of those tests, it also involved a number of problematic aspects that were becoming increasingly evident. For example, in the old model, one difficulty with having the dosage calculation directly linked to clinical courses was that instruction and assessment were quite varied among faculty, often leading to inconsistencies in test content and expectations.

Far more serious than the perceived inconsistencies regarding teacher practice was the fact that each year a growing group of nursing students would ultimately be unsuccessful (even after further re-write attempts) in the math/dosage aspects of the clinical courses, and in some cases would eventually be forced out of the program as a result of two such clinical failures, as per program policy. As one participating instructor shared, “We were seeing probably 8–10 students per year, across all four years, that would fail and would do some sort of remediation, or take some sort of action to be able to continue on in clinical” (P03). 

This perennial situation represented a stressful and costly endeavor for these students, a time-consuming aspect of instructor workload, and a negative reflection on the Collaborative program and the university as a whole. A former Director noted, “If they didn’t pass, they would fail that clinical; if you fail clinical, you don’t pass the year—so that test, that small test, could cost them, you know, a year’s tuition and residence, so maybe $10,000” (P10). Another instructor further shared, “It became problematic, not only for our student population, which we were very concerned about, but our CASN [Canadian Association of Schools of Nursing] accreditation body was not looking at it favourably either” (P02). Departmental discussions began to focus on ways in which the program might consider shifting towards a different type of model, given the student difficulties relating to the math skills assessment.

### 3.2. Transition to the New DCCT Model

**Figure 2:**
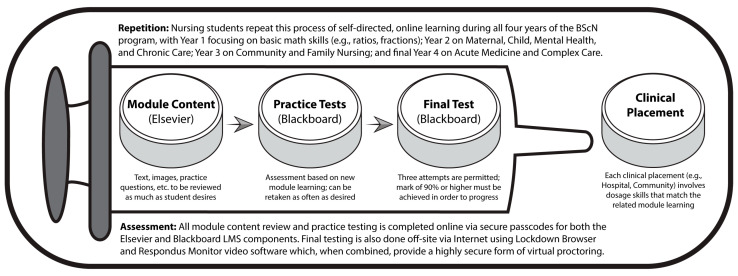
Overview of the new Dosage Calculation Competency Test (DCCT) model.

One key aspect of designing the new model was to carefully analyze the four years of the program in terms of the clinical placements and related math skills and to then create a relevant series of math modules that corresponded to these required skills needed in the different placement settings (Figure 2).

One instructor who was heavily involved in the development of the new model explained this process, highlighting the importance of tailoring the modules to reflect the context-specific math skills.

**P15:** That is when we began developing key spots in the program throughout the eight semesters where the math testing would be building the skills of the student, and constantly getting them to be repetitively learning and retaining the math skills right up until fourth year. Because when they graduate, they have to be able to determine what the right dosage would be to give to any patient. It’s something you learn but can then easily forget—so you have to maintain the skills throughout.

Another vital aspect of the new model was to change the math component (i.e., the final written test) of each clinical course into a separate (i.e., not part of course assessment), required, self-directed activity that would serve as a *prerequisite component* for each subsequent clinical placement. “The decision was made to move to a stand-alone dosage program that is treated like a basic competency, just like having your CPR, or your Non-Violent Crisis Intervention training as requirements for students going into clinical” (P11). An instructor explained: “They would be required to pass each module before they could move on.... If the students didn’t pass any test, they wouldn’t be allowed to go into clinical, however it no longer meant a ‘Fail’ on their transcript” (P01). Another positive result of the new model approach was the overall consistency in the teaching of the math dosage skills: “We are teaching the students the same way to do the math in all four years, as opposed to having different people in different ways each semester, that are covering the same content” (P11). 

Not only would the new DCCT model involve a logical progression of nursing math skills and a clinical prerequisite approach, it would also feature an in-depth collaboration with a software company, Elsevier, and the co-creation of a uniquely tailored online learning and assessment system. One instructor recalled the departmental discussions which led to the new model adoption:

**P02:** I remember many faculty meetings where we spoke about the DCCT at length. We talked about what is the best way to implement this into the curriculum. Do you want to make it a stand-alone course? Will it be worth course credits?... We decided through many discussions that we would purchase this DCCT software and implement it as a stand-alone, pre-clinical requirement.

In the new DCCT model, students are assigned modules essentially in an online textbook that they work through, learning at their own pace. There are deadlines set, and there is access granted to new content each semester. Once they have completed the online modules, then they go into Blackboard (the Learning Management System) and there are practice tests that have similarly worded questions that they’ll see on their final test. Students can take these practice tests as many times as needed to prepare for the writing of the final test for that term. One of the challenges for students with the self-directed nature of the new model was just planning when one would complete the practice work and then write the final test. As one student noted, “It was basically just fitting in into your schedule, when you weren’t working on your five other courses—so, basically your time management skills” (P13). 

The new DCCT model was implemented in stages, with P03 piloting the new system first with her Year 1 and 2 nursing students in Fall term of 2012, and then subsequently with Year 3 and 4 students as they worked their way through the program. “It was during that time that [P03] was doing first and second year, so we started out with one group, and then we did it for third and fourth, and she took on all of them as it went along” (P15). One of the main reasons for staggering the implementation of the model was the cost of the software for students, thus the first group of students would have access to the online software for at least three, if not four years.

### 3.3. Ongoing Modifications to the DCCT Model

Once the new model had been put in place, the department sought continual feedback from both the instructors involved and from the nursing students by way of anecdotal feedback and informal surveys. The instructor who had been pivotal in collaborating with Elsevier in the creation of the new online package reminisced on how the new model very quickly showed promising effects.

**P03:** When I left three years ago [ca. 2017], we did not have one single student who had to be withdrawn from clinical as a result of dosage calculation. We did have a few students who needed to do multiple re-writes, and so we put a remediation package together for them.... They had to go through the Elsevier program again, and all of the practice tests again, and then when I saw that they had passed those, then they would get a third attempt, and all of them passed.

The same instructor had also used an online survey tool, *Survey Monkey* (Survey Monkey, San Mateo, CA, USA) to collect feedback from nursing students in the program: “I did some statistics gathering every single year. I would say that about 90 to 95% who would reply on the survey really much preferred the self-directed ability to test and retest” (P03).

One of the major ongoing modifications to the new DCCT model is related to how and where the actual testing is performed. Chronologically, the model moved from final dosage tests being written by hand in classrooms, to being written on a computer within a lab proctored by an instructor or designate, to ultimately being written off-site using the Internet and a combination of security programs in order to prohibit or reduce potential cheating. The Faculty Lead compared the previous and current DCCT models.

**P11:** There’s a limitation on campus with the number of computer labs, and number of computers in each lab, and so it was very labour-intensive to have 300 students write tests on-site.... We’ve changed it so that they have the flexibility to write it when they’re ready.... and starting this academic year [2019–20], they test at home.... We use *LockDown Browser* as sort of the basis, then in first year, second semester they also use *Respondus Monitor*, a video proctoring software.

Students expressed a number of issues that they found stressful regarding the move to the fully online, off-site version of the final testing. First, they felt stressed about having to find an appropriate space to write it, often having to leave their homes or residence spaces to do so. As one student shared, “I live in a very hectic and very busy household, so I actually had to find a space in my community at the local library where I could go to write my test, that had a good internet connection” (P07). The Faculty Lead noted that an attempt was made to help students with this challenge by arranging for library study rooms and vacant former office spaces in the university to be used for potential quiet writing sites. Second, some students missed having a proctor and a technician present during the test writing in case they had questions or experienced technical issues, respectively. Third, the web camera software requirements were felt to be somewhat overly restrictive in terms of non-applicable warnings automatically issued by the software during testing. Fourth, students with special needs and related accommodations initially encountered certain challenges with the move to online, distance evaluation but these access/timing issues were ultimately addressed by the Faculty Lead.

In contrast, many students expressed an appreciation for online, off-site testing, highlighting the reduced amount of travel time as one of the main benefits of the new model. As one such noted, “Personally, since I live outside of [city], it has taken me, due to the weather, upwards of 40 minutes to get to school, and sometimes I didn’t even spend that long writing the test” (P05). One Year 3 student highlighted a perceived parallel with the self-directed module work, “I thought it was less stressful because you’re more independent that way. You’re used to doing your dosage math on your own, and there’s no strict time to come and write it, so I appreciated being able to do it online” (P09). Faculty members were originally hesitant and cautiously reluctant to move towards an Internet-based assessment process for fear of potential student cheating. However, with the advent of better software options, such as *LockDown Browser* and *Respondus Monitor* (Respondus, Redmond, WA, USA), they ultimately became more at ease with the system. 

## 4. Discussion

In this section, we will address Research Question 2 by exploring further themes that emerged from the case study data dealing with student and instructor perceptions of overall DCCT model effectiveness in terms of mathematical confidence and competency.

### 4.1. Four Types of Student Learning Associated with the DCCT Model

Based on the survey data and interview transcripts, several different types of learning relating to the new DCCT model became evident. Three of these were arguably part of the planned curriculum, while a fourth seemed to develop in a more informal, spontaneous manner.

Perhaps the most distinctive feature of the new DCCT model was the *self-directed learning* aspect of both the module-based math content learning and of the assessment experiences. Nursing students were free to work on the content at their own pace each term, and to complete both the practice tests and final test for each clinical course whenever most convenient for them, within the allotted timeframe windows. This theme became evident in the survey open-response item and was further elaborated on by participants during the interview process.

When asked about what they perceived as the most beneficial aspects of the dosage course experience, many of the 44 survey participants focused on their appreciation of the flexibility surrounding the self-directed, independent nature of the DCCT module learning. For example, one student noted, “Being able to complete at my own pace, and I can focus my studying on places I need extra practice.” Another wrote, “I am glad that we were able to learn this information on our own time and not have to sit in a lecture to learn it.” One first-year student reflected on student procrastination:

**P13:** It’s really based on your time management skills as a student. This program is so time-consuming with clinical, which has the lab, as well as the other four courses. And then going to the hospital or long-term care facility as well, so it’s really hard to fit it all in.... It’s not like it’s super draining or demanding, but it’s just setting it up at the beginning of the semester to do it throughout the year, and not leaving it until the very end.

Nurse instructors indicated that they too felt that the self-directed aspect of the new DCCT model was important for their students, in terms of the perceived benefits during both the undergraduate experience and throughout their future nursing careers. One faculty member noted, “I think what is working well is that it allows students who are strong to go ahead and work independently” (P03). Another colleague explained: “I think it’s good. I like the fact that it’s not attached to a clinical. I think for students it’s important for them to develop that skill of doing self-directed learning, because nursing is a life-long learning profession, and I think that they need to be encouraged to do that” (P01). According to Faculty Lead, it also allows for adequate time for study.

**P11:** I think one of the strengths is that it allows the students to take as much time as they need, and sort of adjust their studying to when it’s good for them. I’ve had students who have really, really struggled with their dosage, and who will contact me in January to start working through things, but then also students who are stronger who don’t need to take as much time, and they’ll start reviewing the modules only a few weeks before.

Beyond the self-directed aspect of the DCCT model, students were also presented with a *personalized learning* experience within the Elsevier platform itself. By this, we mean that the software was designed to present dosage calculation questions that featured several different solution methods or strategies. Early on in each module, students were asked to try the various methods and to select the one that they found most easy to follow and replicate when solving similar problems. The software would then proceed to present all subsequent module content and related questions/solutions using only, or predominantly, that selected method for sake of consistency. As one student noted, “There’s like a bunch of different ways you can do it in the online modules, there’s like three different formats for a lot of these questions, so it was finding which one works for you best” (P06). Another likewise explained:

**P12:** It offers you four or five different ways to complete the same question, depending on your learning style.... It’s set up so that it presents ways to approach each of those questions with different math styles, like proportional, or dimensional, or ratio analysis. The idea is that you’re going to go through and you’re going to learn all four different ways first, and then you’re going to decide which one works best for you.

Thus, even though the module format was consistent and the content was being presented in a standardized and repetitive way throughout all four years of the program, there was also the ability to personalize the learning.

A third type of learning experienced by students in the new DCCT model was that of *contextual (or embedded) learning*. While this aspect had indeed been part of the previous DCCT model with math skills being taught by the clinical instructor in a classroom as part of the overall course delivery, in the new model the clinical lab instructors expected nursing students to have already encountered certain pertinent math skills as part of their self-directed learning progress within the Elsevier/Blackboard system. Of course, the success of such an educational approach requires that (i) students actually take the time to complete the related learning prior to the group session; and (ii) that instructors plan their courses carefully to ensure that students are aware of, and are given adequate time to complete, the new independent learning content prior to each simulation lab session. 

When both of these conditions were met, and the lab instructors presented math concepts or skills within the context of a clinical simulation lab activity, participants indicated that the resultant learning was usually very positive as it was seen as directly relating to practice. One student shared, “We learned about calculating primary, secondary, and continuous drip calculations in the lab, and then at the end, we had to get individually tested on it” (P09). A Year 2 student likewise noted, “I found that there was quite a consistency between what we learned in the lab and what we had to do for calculating dosage” (P12). Another participant shared: “For things like basic drug calculations, we did have to do them in clinical class and we also did that in the lab, which is really neat, and we had evaluations based on that” (P13). A final year student reflected back on this form of contextual learning:

**P07:** I liked it when our instructors incorporated it into our labs, because we are learning the skills and so allowing you to integrate the dosage into it was very similar to simulating what you would have to do if you were to encounter that situation on a hospital floor, or when working as a nurse.

Faculty mostly agreed with the value of embedding dosage skill teaching within authentic contexts that allowed for the direct application of the new learning. The former Dean noted, for example:

**P10:** I don’t think you can teach anything without context—that’s part of my own teaching philosophy. You know, you can theoretically do this stuff, but you need to use real experiences, or use case studies, which are great and safe contexts. So, I mean, you should be teaching the math skills kind of theoretically, but you have to push it into a practical context because nursing is nothing but, you know, practical knowledge.

However, in instances when either one or both of the above-mentioned conditions had not been met, this sometimes led to less than satisfactory results, for a number of reasons. For example, a Year 3 student recollected: “I remember they were saying things like, ‘So, you guys know how to do this, right?!’” (P09). Another stated, “I think it’s important, but I found that when they tried to integrate dosage questions into labs, they would go very fast and skim over it because it was expected that you had already done it by yourself, and so would understand it” (P14). In terms of why this mismatch may have been occurring, students hypothesized that there may have been a lack of communication between the Faculty Lead and the clinical lab instructors in terms of the dosage module content and related deadlines. 

Given the very busy schedule of a nursing student, participants noted that it was often difficult to find time within one’s school, clinical, and general life event schedules to complete the DCCT modules, practice tests, and final test. Students shared that having opportunities to access the online material earlier (e.g., summer break, or prior to the beginning of a given term) might have made things easier for them in terms of learning the math skills in advance of the corresponding lab simulation or class sessions. A first-year student noted: “I’m pretty sure they’re opening our dosage course up in the summer for next year, so that’s going to be really nice to get it all done in the summer. Definitely beneficial... when you don’t have everything else to do” (P13). A senior student concurred: “I think it would be very helpful, yes, especially for second, third, and fourth years. I know if I had that option available to me, I would have used it in terms of doing it before courses started” (P14).

A fourth and final type of learning relating to the new DCCT model that was discussed by participants in the interviews was that of informal, *social learning*. In other words, although the primary mode of new math skill learning was independent and self-directed using the Elsevier portal and Blackboard LMS, it was not uncommon for students to make their own arrangements to review DCCT material with others, in pairs or small groups. All of our student participants described having experienced or observed this kind of spontaneous and informal social learning behavior while in the program. This would take place on-site, in available classrooms or library study rooms, or off-site using email or social media. A Year 1 student describes this kind of informal social learning phenomenon:

**P08:** If somebody would say, “I’m really struggling with long division,” or something, then we would all start talking about that topic.... It was just whoever had already done the modules, or who understood it the best, would just explain it to everybody else, and if multiple people understood it, then they would just kind of break off into smaller groups and talk one-on-one with somebody.

Upper year students (Years 3 and 4) recalled similar experiences as they reflected back on their social learning, “We went to the library and were together every single time. So, definitely a social setting for curriculum review, of working together, and understanding it together, and figuring out what the requirements were” (P06). Another noted:

**P04:** Yes, we would normally go get a room at the library and just study there together.... I think that group learning is probably the best way for nurses to learn. Say you have four people in a group, and you might have one person who feels super confident with the math, like they know what they’re doing; then you have another person who may be as confident, but doesn’t really know how to explain things to others; and then you’ll have one or two other people who have no idea what’s going on, and are just trying to stay above water.

Instructors indicated an awareness and general approval of this kind of informal social learning that was happening within the new DCCT model. “It’ll naturally happen, as that’s the way nursing students study, they tend to do a lot in groups, even for their final NCLEX [National Council Licensure Examination-RN] exam. Yeah, I think that’s great, and I have no problems with that” (P10). 

In summary, nursing students experienced self-directed, personalized, contextual, and social learning—the first three types being directly related to the planned curriculum, and the fourth type developing more spontaneously.

### 4.2. Student Perceptions of Mathematical Confidence/Competency

Students who took part in the online survey shared mixed responses in terms of their perceptions of the overall effectiveness of the Collaborative program in adequately preparing them for the math realities of nursing practice.

**Figure 3:**
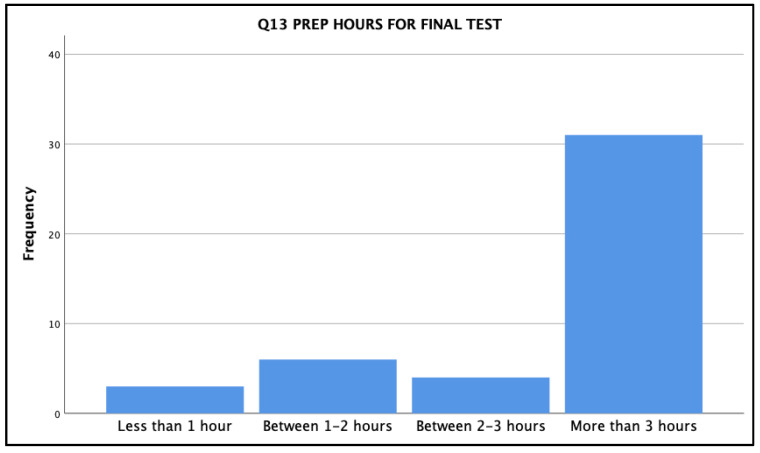
Number of hours, on average, spent on preparing for final dosage test.

In the online survey, when asked about the number of hours that nursing students had spent, on average, in preparing for the final qualifying test each term, the following data were shared (Figure 3). 

Thirty-one of the forty-four participating students indicated that they spent more than three hours in preparation for this final assessment. Clearly, it was something that was of importance to them, given the required 90% achievement in order to proceed to the next clinical placement the next term.

**Figure 4:**
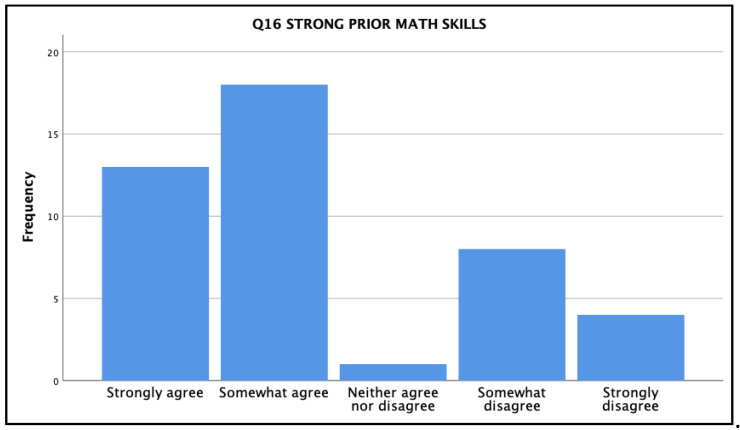
Q16 survey results regarding participants’ perceptions of their own math skill strengths reflecting back on when they entered the program.

When asked in Q16 if they felt they possessed strong mathematical skills prior to starting the nursing program, students noted a range of responses (see Figure 4). Thirty-one of the forty-four (70%) students indicated that they either strongly (13) or somewhat (18) agreed with this statement; while 12 (27%) noted they somewhat (8) or strongly (4) disagreed with the premise.

When asked if they felt that the dosage program has helped them with their understanding of medication administration, 28/44 (64%) indicated a strong (6) or somewhat (22) agreement; while 10/44 (23%) noted a somewhat (8) or strong (2) disagreement, with 6/44 (14%) being undecided. In Question 18, we asked participants if they believed the dosage program has helped them with overall math skill development. Results here indicated that 21/44 (48%) indicated a strong (4) or somewhat (17) agreement; while 14/44 (32%) noted a somewhat (10) or strong (4) disagreement, with 9/44 (20%) being undecided. 

In terms of perceived content alignment, Question 20 in the survey presented the following statement, “The mathematical content covered in the dosage course aligns well with the knowledge and skills that I will need in the clinical setting” Results showed that 27/44 (61%) of survey respondents indicated a strong (3) or somewhat (24) agreement; while 11/44 (25%) noted a somewhat (8) and strong (3) disagreement, with 5/44 (11%) undecided. 

The final two Likert questions in the survey focused on perceived readiness for practice. Question 21 proposed that, “I was able to use the math skills learned in the dosage program in my clinical area in a timely manner (i.e., I felt confident with my math skills in order to perform related tasks)” Feedback from participants indicated that 20/44 (45%) indicated a strong (8) or somewhat (12) agreement; while 9/44 (20%) noted a somewhat (6) or strong (3) disagreement, with 15/44 (34%) undecided. Question 22 stated more specifically that, “The dosage program, in combination with my clinical courses, make me feel confident with my ability to safely give medications in the clinical setting” Here, survey respondents indicated that 24/44 (55%) were in strong (8) or somewhat (16) agreement; while 5/44 (11%) noted a somewhat (5) or strong (0) disagreement, again with 15/44 (34%) undecided. 

Survey responses regarding perceived benefits of the dosage calculation program included a number of comments regarding positive curricular alignment. One nursing student shared, “Dosage gave me the opportunity to practice a lot of basic math that I had forgotten about, as well as learning and memorizing new and helpful conversion factors” Another noted, “I find that the questions have real world applications, and that I can see how they apply to clinical practice” “Having case scenario questions that model real-life clinical situations” explained one participant. Yet another student noted: “It allows time for students to specifically focus on dosage calculations before the test, rather than having it integrated within a course and potentially seeing it drowned out by other skills” “It was a good insight of what is to come in the nursing profession, when it came to medication” stated another participant. “The number of ways medications can be administered (i.e., dosages, different unit conversions, titrations, routes) was covered” noted a fellow student, “helping us learn how we would give medications in the clinical setting” One student highlighted needed skills, even in light of the reality of the pharmacy’s role in drug calculations.

It is helpful because it introduces what kind of calculations are needed in dosages. It teaches what to expect in the clinical setting. Although pharmacy does most dosage calculations, nurses need to understand them and be able to calculate I-V rates, especially mg/hr versus ml/hr, on their own, confidently. Students find IV calculations the hardest to understand but they are used in most areas of practice and the nurse is usually responsible for calculating it. 

So, while a majority of survey participants felt that they were mathematically competent when reflecting back to their point of entry into the program (Q16), and felt that the DCCT model was helping their understanding of medication administration (Q17) as well as featuring a curriculum that was well-aligned with the realities of practice (Q20); less than half of the surveyed group felt confident in their general math skills in clinical (Q21), and only slightly more than half felt confidence in their ability to give medications while on practicum placement (Q22). Keep in mind that this is a relatively small sample size (approximately 10%) of current nursing students and that we have not teased apart the responses by program year. Notwithstanding, we are curious to know *why nursing students who feel they were mathematically competent when reflecting on their math skills upon program entry, and who are passing final dosage tests with 90% or greater achievement each term, still do not seem to feel overly confident about their related math dosage skills*. Perhaps it has something to do with the high-risk nature of actually administering medications to real patients in healthcare/community contexts? Is it possible that some, perhaps many, students are barely passing the dosage tests via persistent efforts and multiple attempts, and hence still do not feel overly confident about their math dosage competency?

Much of today’s medication dosage calculations are carried out by a pharmacist within the hospital, and medication capsules are often prepared and delivered to the nurse through an Automated Dispensing Cabinet (ADC). Furthermore, although much of any remaining calculating work is carried out by the nurse with a calculator, computer, or mobile phone, instructors and their students still understand the importance of critical thinking and the ability to be able to estimate and check answers for reasonableness. A Year 1 student shared her clinical observations:

**P13:** A lot of the healthcare facilities have automated dispensers, where all of the medication is pre-packaged... and they use the barcodes on patients’ wrists, and those on their medications to match up patients with the already prescribed pills. So, the nurse doesn’t really have to calculate.... With experience you’d be exposed to more and more, so you’d know the reasonable ranges without having to calculate it every time, but obviously it is still important to know how to do it.

In our previous research, we called for the introduction of mobile smartphones and Internet resource searching to become part of all nursing education programs, given the commonplace reality of these activities in the nursing profession [21]. The use of mobile phones by nursing students in clinical contexts, and by practicing nurses as observed by our students during these placements, once again presents an interesting point of discussion. While smartphones are often formally banned for students during clinicals, and sometimes even prohibited by hospital policy for nurses, their use appears to be widespread and varied in nature. In this study alone, the reported uses of mobile phones by nurses included the following seven items: (i) math calculations; (ii) medical content searching [conditions, treatments, symptoms]; (iii) as a timer; (iv) checking patient vital signs with new apps/tools; (v) prompts/notifications for patient medications using hospital-provided software; (v) language translation, especially in high-immigration centers; (vi) communication/social networking; and (vii) personal safety (e.g., breaks, parking lot at night). One student noted, “I have not ever in my four years seen a nurse calculate with pencil-and-paper—I have always seen them use a calculator” (P07). When asked about technology use (smartphones, Internet) within nursing practice, two other participants shared the following observations:

**P04:** Everyone carries a cell-phone. It’s like it’s essential now to carry your cell-phone as a nurse.... If something comes up and a doctor is talking, and they say something that you’ve never heard of before, you can quickly Google it instead of looking stupid.

**P06:** Every nurse I’ve seen in practice has their cell-phone on them, using it for dosage calculations or to look up medications.... One of the applications that is loaded onto all of the hospital accounts is called *UpToDate*, where it has all of the information, like medication administration, processes, and common symptoms.

**P07:** I have not ever in my four years seen a nurse calculate with pencil-and-paper—I have always seen them use a calculator.... There’s a calculator at their dosage station, but for emergency situations they might have their phone in their pocket, and so they would just pull that out and use that as a calculator.

However, not all participants observed smartphone usage among nurses and students on clinical placements. One student noted, “I know for students it’s not allowed. At my long-term care home placement, they also weren’t allowed to have cell-phones at all on the floor. So, I haven’t seen anybody with any cell-phones or using them—even the staff there weren’t allowed to do it” (P08). Another stated, “You weren’t supposed to have your cell-phone on you. I think some nurses would use it as a calculator, but I think that was it. It was pretty strict... I didn’t really see a lot of use of cell-phones, actually” (P09). Clearly, this is an area requiring further research in terms of curriculum reform.

When asked in the interviews about their perceptions of overall preparedness for practice based on their experiences with the new DCCT model, nursing student participants by and large appreciated the program. One Year 3 student shared her thoughts:

**P09:** Being tested yearly, it makes me feel like I’m being kept up-to-date.... I think [university] has a really good reputation for making good nurses, and it’s because of the way that we test here. It holds everybody accountable.... My friend, she hated dosage calculations—but I think, deep down, she does appreciate having to do it each year, because now she’s good at it.

Another Year 4 student summarized her undergraduate experience: “I feel well-prepared. I’ve also been able to kind of seek out learning opportunities on my own, outside of the program, like in summer employment, and kind of be exposed to building confidence in those dosage situations” (P07).

Like any program structure or model, there is always room for improvement. Student participants in both the survey and the interviews shared a number of criticisms in this regard. First, some noted that there was a mismatch between module practice and final tests. Some students also experienced problems with the software interface itself, or encountered issues relating to internet access or connection stability. 

### 4.3. Instructor Perceptions of Mathematical Confidence/Competency

Faculty members invariably shared how that they felt mathematical skills were essential for nursing practice, particularly with regard to computational competency (with and without technology), as well as an ability to use critical thinking to estimate answers and to analyze the reasonableness of answers. The former Dean noted as follows:

**P10:** One is just knowing your basic math skills—so, whether or not you know two plus two equals 4, and then being able to check an answer for reasonableness. You also have a legislated responsibility to know how much of a drug should go into a patient, whether you received that from the pharmacy through a dispensing machine or prepared it yourself, and whether or not you use a calculator or not, you have a legal, ethical responsibility to know that it’s the correct drug, going to the correct patient, at the correct time.... Yes, even if you have a calculator, I mean, a human has still got to enter the numbers? So, go ahead and get your phone out, and do 25% of 37, you know, I would do that with a phone too, but I have to at least be able to go, “Yeah, I think that answer is correct because 37 is close to 40, so a quarter of that has to be just less than 10.” They should be able to know that.... And you know the bottom-line consequences, if you don’t know how much, or why, and you give someone the wrong drug, well, you’re legally responsible for that, and you could go to jail.... Human error is huge in the healthcare system.

According to another instructor, a nurse must be able to accurately calculate dosages, check answers for reasonableness, and avoid a general over-reliance on technology.

**P15:** Just because a machine says it’s so, doesn’t mean it’s so. You have a 12-lead ECG, and some of those machines interpret what the findings are, but being a Critical Care Nurse and an expert in reading 12-lead ECG’s, I can tell you the ECG machine is wrong all the time.... In terms of the ADS machines, they have not always prevented medication incidents, and in fact, in some cases, they’ve created them.... You still have to open the drawer, check the med cart for what the patient is supposed to be getting at that point and time.... The meds aren’t necessarily going to be in the exact perfect dosage amounts either—they may need to be split, or you’ll have to give two instead of one, because they only come in 25 mg and you have to give 50, three times a day. If you can’t figure that out, then you’re in trouble.

When asked if they felt confident that the new DCCT model was graduating nurses who are competent and confident in their dosage administration abilities, one instructor shared the following summary:

**P15:** The new system provides an opportunity to do some self-directed learning, to really take some ownership and accountability for learning, and to be able to do it at their own pace in an environment of their choosing.... I think that this approach has reduced the anxiety overall, and gives them a repetitiveness that’s often required to retain these kinds of things.

Participating instructors also provided input on what types of changes would be helpful as the DCCT model continues to be monitored and revisited in the coming years. Similar to the student comments, faculty highlighted the following areas: (i) formalizing contextualized, yet unassessed, dosage teaching in the labs; (ii) updating DCCT questions to reflect any changes in nursing practice; (iii) producing short instructional videos that nursing students could play, pause, or rewind as needed; (iv) reviewing the timing of the module releases (e.g., pre-course access); (v) considering a second designated person to help support the complex work of the Faculty Lead; and (vi) increasing communication with Clinical instructors. 

Our case study findings, here presented, offer significant insights into student and instructor perceptions, particularly surrounding the delivery of online curriculum (e.g., modular-based content; a chance for repeated review with achievement thresholds) and assessment (e.g., self-directed testing; online, distance, secure final assessments) methods. These considerations are particularly relevant during the pandemic era since almost all education sectors were required to make sudden transitions to online programming, thereby encountering many of the same challenges, and hence discovering, by virtue of necessity, a number of useful strategies and systems that are worth sharing more widely.

Much research was carried out in the area of improving the mathematical literacy of nurses in terms of programs and strategies used in the undergraduate curriculum. Existing in-depth literature reviews in this area provide extensive background information [22,23]. Qualitative, quantitative, and mixed-method research study designs were implemented to further explore the challenges associated with increasing the mathematical competency [24,25,26,27,28,29] and dosage administration skills [30,31,32] of nursing students. What we add to this ever-growing body of work is a detailed case study of the adoption of a new model that involves a fully online, self-directed, pre-requisite approach. All three of these qualifiers are significant since the fully online aspect addresses current realities in educational contexts; the self-directed aspect allows for maximum student choice/flexibility in terms of their individual learning styles; and the pre-requisite aspect provides for a logical system of progression carefully tailored to mathematical skills related to each stage of the nursing student’s new learning, both academic and practical. 

As we conclude the paper, we would like to suggest some key recommendations for faculty and administrators who may be facing similar challenges surrounding required mathematical/dosage learning. These recommendations are based on the nature of the emergent themes within the case study, and specifically touch on what appear to be priority focal points for both the instructors who were involved in designing and implementing the model and the nursing students who experienced the model first-hand.

## 5. Conclusions

In closing, based on what we perceive as strengths of the examined DCCT model, we would like to share the following seven focal points, or key recommendations, for nursing education programs as they consider the development of similar curricular programming.

1. Focus on Calculation and Critical Thinking: Nursing practice invariably requires both mathematical and dosage calculation skills, as well as critical thinking skills such as mental estimation and the ability to check answers for reasonableness. Instructors were unanimous on this point, all indicating that nurses must possess both hand-written and technology-based dosage calculation competency.
2. Focus on a Mandatory Prerequisite Structure: As opposed to a model wherein the dosage calculation component was a composite and assessed feature of clinical courses, dosage testing/competence is now also a prerequisite for each clinical admission. Instructors felt that insisting on this requirement helped students to understand the critical nature of dosage skills, and to increase their readiness.
3. Focus on Practicum-Relevant Skills and Repetition: The dosage modules, involving content, practice questions, and tests, are purposefully aligned with relevant practicum placement skills, and students complete dosage modules throughout all four years. For example, Year 1 focuses on basic math skills (e.g., ratios, fractions); Year 2 on Maternal, Child, Mental Health, and Chronic Care; Year 3 on Community and Family Nursing; and final Year 4 on Acute Medicine and Complex Care. The majority of both students and instructors consistently spoke to the importance of repetition, throughout all four years, as being key to learning.
4. Focus on Self-Directed, Personalized, Embedded, and Social Learning: Module learning is self-directed, personalized via selected solution methods, reinforced through demonstrations within the simulation labs, and often supported by informal peer-to-peer tutoring. The majority of participating students emphasized their appreciation of a self-directed program that allowed them to review content as many times as needed, and whenever most convenient to do so. Instructors stressed the importance of connecting the module dosage/math learning to nursing practice by way of the corresponding simulation lab experiences and were also not surprised to find students informally drawn together for purposes of review.
5. Focus on Online Access and Secure Assessment: All dosage module work is completed online through secure access to the Elsevier Evolve portal and Blackboard LMS, including the final test using *LockDown Browser* and *Respondus Monitor* software. Perhaps the most important aspect of the new DCCT model, in light of current trends in online education, was the complete transition, over time, from onsite, computer lab-based assessment to secure, online, assessment practices including final tests. While both instructors and students shared initial reservations about such a transition, the majority of participants indicated that the benefits of such an approach, once established, far outweighed the previous model.
6. Focus on a Designated Administrator and Student Supports: A Faculty Lead, through the designated workload, is strategically positioned to oversee the DCCT model, tracking all student progress, addressing technological/content issues, and providing student support. Instructors recounting the planning and implementation of the new DCCT model all stressed the importance of clearly establishing a recognized position in which a faculty member would feel well supported by the department, and from which they could offer student assistance.
7. Focus on Cyclic and Data-Driven Program Evaluation: Ongoing review of the DCCT model is conducted through regular student surveys, student achievement data analysis, and faculty/clinical instructor input that is shared via email and at departmental meetings. All new programs or models need to be revisited and revised according to ongoing and careful review. Instructors in the study relied heavily on both formal (surveys) and informal (anecdotal) feedback from their students, and this information shared in departmental meetings, guided progress.

Mathematical competence and confidence are vital aspects of contemporary nursing practice, even within an era of increased technological advances. Nurses must be able to complete dosage calculations, with and without the use of digital machines, and to be able to judge dosage calculations for reasonableness. In preparing nurses for this complex profession, undergraduate nursing programs must find a way to address these math- and technology-related competencies in a way that is both pedagogically sound and pragmatically sustainable. The online, self-directed, prerequisite DCCT model that was examined herein, arguably provides just such an approach for nurse education programs.

**Clinical Resources**

▪ *Blackboard Learn*: An Advanced LMS
  https://www.blackboard.com/teaching-learning/learning-management/blackboard-learn (accessed 11 December 2021)
▪ *Elsevier Dosage and Solutions*, Online Textbook Resources
  https://www.elsevier.ca/ca/specialty.jsp?lid=4&sid=636 (accessed 11 December 2021)
▪ *Respondus Monitor & LockDown Browser*: Assessment Tools for Learning Systems
  https://web.respondus.com/he/monitor/ (accessed 11 December 2021)
▪ Wolters Kluwer *UpToDate* Clinical Decision Support Resource Software
  https://www.uptodate.com/home (accessed 11 December 2021)

## Data Availability

As per Canadian Tri-Council policy, all survey and interview data will be kept on the researcher’s secure computer for a minimum of five years, and after that, deleted.

## Appendix A. Survey Questions

Q01. Program of Study: Please select the appropriate program identification.
Q02. Current Year of Study: Please select your current year of study in the nursing program.
Q03. Gender: Please indicate your gender.
Q04. Age: Please indicate your current age bracket.
Q05. I encountered ‘glitches’ and problems with the purchased Elsevier online learning modules:
Q06. I encountered ‘glitches’ and problems with the Blackboard component of the dosage course:
Q07. I required technical assistance to maneuver through the purchased Elsevier online learning modules:
Q08. I required technical assistance to maneuver through the dosage course:
Q09. I have accessed additional help to understand the math content from (select all that apply):
Q10. Help was readily available when I requested it from the Faculty Lead instructor:
Q11. Help was readily available when I requested it from the Learning Systems Technologist:
Q12. I found the independent and self-directed nature of the dosage program to be:
Q13. On average, how many hours of studying does it take you to prepare for a DCCT final test?
Q14. I found the time allotted to prepare for each module to be:
Q15. I found the time allotted to write the actual DCCT test each year, in general, to be:
Q16. Prior to starting in my nursing program, I felt I had strong mathematical skills.
Q17. I believe the dosage program has helped me with my understanding of medication administration.
Q18. I believe the dosage program has helped me with my overall math skill development.
Q19. The Blackboard quizzes that prepared me to write the final DCCT test were:
Q20. The mathematical content covered in the dosage course aligns well with the knowledge and skills that I will need in the clinical setting.
Q21. I was able to use the math skills learned in the dosage program in my clinical area in a timely manner (i.e., I felt confident with my math skills in order to perform related tasks).
Q22. The dosage program, in combination with my clinical courses, make me feel confident with my ability to safely give medications in the clinical setting.
Q23. In your opinion, what has been the most beneficial aspect of the dosage course experience?
Q24. In your opinion, what has been the most challenging aspect of the dosage course experience?
Q25. Please use this open response box to share any further comments you may have about your overall experience with the dosage course material, related testing, instructor/technologist supports, etc.? Please be specific about the year or years of the program you are discussing.

## Appendix B. Interview Questions

Nurse Instructor/Administrator Interview Questions

1. How long have you been associated with the Nursing Program/School at Nipissing U?
2. Can you please describe your recollection of how the mathematics component of the undergrad nursing program was structured in the past (if applicable), and then also how and why it was changed with the introduction of the mandatory, non-credit, self-directed DCCT program?
3. What observations have you made regarding the nursing student attitudes and skills relating to the mathematics required for nursing throughout the past decade (before and after the change)?
4. In the current DCCT system, what strengths do you think support the use of this approach?
5. In the current DCCT system, what areas of improvement do you think might be considered?
6. In your view, how prepared are the graduating nurses for the NUCLEX and nursing practice in terms of their required mathematical skills and confidence relating to these skills?
7. Do you have any other comments that you’d like to share relating to mathematics for nursing?

*Nursing Student Interview Questions*

1. Based on your survey/questionnaire comment, [selected survey quotation deemed of further interest to the research team], can you please provide us with more information regarding your perceptions on this first issue.
2. Based on your survey/questionnaire comment, [second selected quotation deemed of further interest to the research team], can you please provide us with more information regarding your perceptions on this second issue.
3. Do you have any other comments that you’d like to share relating to mathematics for nursing?
